# Flexible positioning of a large area detector using an industrial robot

**DOI:** 10.1107/S1600577522006300

**Published:** 2022-06-27

**Authors:** Christina Reinhard, Michael Drakopoulos, Christopher M. Charlesworth, Andrew James, Hiten Patel, Paul Tutthill, Davide Crivelli, Hans Deyhle, Sharif I. Ahmed

**Affiliations:** a Diamond Light Source, Harwell Science and Innovation Campus, Didcot OX11 0DE, United Kingdom; b NSLS-II, Brookhaven National Laboratory, Upton, NY 11973, USA; c YASKAWA, Walworth Road, Newton Aycliffe DL5 6XF, United Kingdom

**Keywords:** synchrotron power diffraction, robot, stability, repeatability, SR-XRD, automation, detector positioning

## Abstract

The DIAD beamline for Dual Imaging and Diffraction at Diamond Light Source has opted to use an industrial robot to position its Dectris Pilatus 2M CdTe diffraction detector. This setup was chosen to enable flexible positioning of the detector in a quarter-sphere around the sample position whilst reliably holding the large weight of 139 kg of detector, detector mount and cabling in a stable position. Metrology measurements showed that the detector can be positioned with a linear repeatability of <19.7 µm and a rotational repeatability of <16.3 µrad. The detector position stays stable for a 12 h period with <10.1 µm of movement for linear displacement and <3.8 µrad for rotational displacement. X-ray diffraction from calibration samples confirmed that the robot is sufficiently stable to resolve lattice *d*-spacings within the instrumental broadening given by detector position and beam divergence.

## Abstract 2

An industrial robot is being successfully used to position a diffraction detector system at the DIAD beamline at Diamond Light Source. Weighing a total of 139 kg, the detector system can be positioned with a linear repeatability of <19.7 µm and a rotational repeatability of <16.3 µrad. The detector position stays stable for a 12 h period with <10.1 µm of movement for linear displacement, and <3.8 µrad for rotational displacement.

## 1. Introduction

Removing technical restrictions from experimental geometries gives synchrotron researchers more freedom in choosing optimum conditions for their measurements.

For powder- and single-crystal diffraction experiments, new technology requires (i) the use of new detectors, providing larger detection areas and therefore becoming bigger and heavier, and (ii) the need to position these detectors in multiple positions, often adding significant complexity to the remainder of the endstation.

One idea to overcome some of these limitations is to employ industrial-scale robots in the endstation design as a detector positioning system, allowing to hold large detectors precisely at multiple positions whilst increasing accessibility within the endstation.

With robots already being used regularly at synchrotron beamlines for sample manipulation for many years, they are now also increasingly used for detector positioning purposes.

While there are concerns about the use of robots for detector positioning purposes regarding the mechanical suitability (stability and positioning repeatability) as well as around safety during user operation, robots open up new opportunities due to their flexibility. Some instruments have already implemented robots for the positioning of diffraction detectors, such as NanoMAX at MAX IV (Bring, 2018 ▸; (Johanson *et al.*, 2021 ▸) and MFX at SLAC (Sierra *et al.*, 2019 ▸); others are in the process of implementing similar systems, such as NANOSCOPIUM at SOLEIL (Abiven *et al.*, 2019 ▸). These positioning systems so far carry detector systems with a relatively small load of <25 kg.

The DIAD beamline for Dual Imaging And Diffraction at Diamond Light Source (Reinhard *et al.*, 2021 ▸) also opted to use an industrial robot arm to position its diffraction detector. By placing the Dectris Pilatus 2M CdTe detector (49 kg) using an industrial robot arm, the detector can be positioned almost anywhere within a quarter-sphere around the sample position, minimizing many technical restrictions and allowing for creative application of diffraction methods. In total, the detector plus mounting provision and cables weighs 139 kg.

The need for flexible detector positioning becomes clear when looking at the variety of research to be conducted on DIAD. Research areas from material science to biomedical studies require access to various diffraction geometries to optimize data quality. Metallic specimens often exhibit a strong texture. For example, in phase analysis of metallurgical samples or chemical processing, one needs a wide coverage of reciprocal space at medium resolution, whereas, for strain analysis, a higher *q*-resolution for selected reflections may be desired. In the future, the user community will also explore more unusual diffraction modes such as fibre diffraction, requiring access to smaller momentum transfer, for bio­medical research or for composite materials.

Given the demand for a wide coverage of *q*-space, the beamline operational X-ray energy range of 7–38 keV and the size of the Dectris Pilatus 2M CdTe detector, the detector positioning needs to be flexible and allow access to both forward and backward (>90°) scattering angles.

In this paper, we will present data on the mechanical stability and repeatability of a detector positioning system based on an industrial robot using various metrology measurement techniques. As the final performance of the system can only be judged from the X-ray diffraction data, a comparison between the metrology and X-ray data will be presented.

## 2. System specification

**Figure 1:**
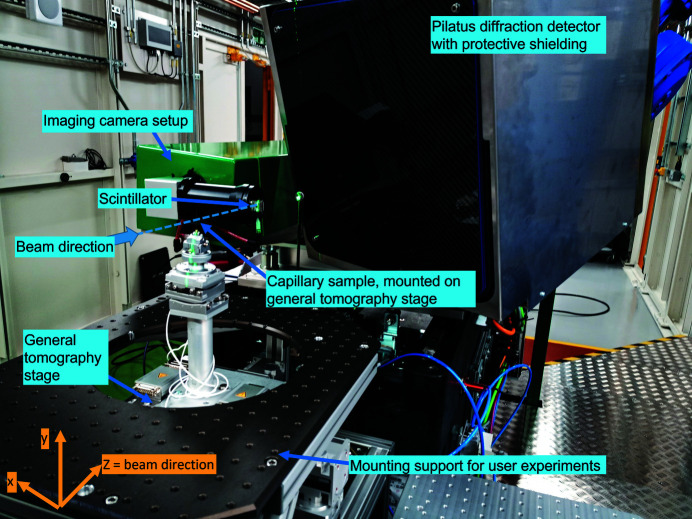
DIAD endstation with capillary sample mounted on the general tomography stage, the imaging camera setup and the Pilatus diffraction detector.

As DIAD is designed as a beamline to provide imaging and diffraction techniques, the DIAD endstation provides setups for both X-ray imaging and diffraction next to each other for quasi-simultaneous data collection from a sample. This design concept required the imaging camera to be positioned in the direct beam position. Therefore, restrictions apply to the diffraction geometry; for example, in the forward scatter direction where the direct beam position is blocked by the imaging camera (see Fig. 1 ▸). For simultaneous diffraction and imaging experiments, scattering angles above 10° can be captured by this setup.

Despite these restrictions, the Dectris Pilatus 2M CdTe detector can be positioned to cover most of the upper reciprocal quarter-sphere around the specimen with typical sample-to-detector distances in the range 300–600 mm. For non-standard experiments without simultaneous use of the imaging setup, larger sample-to-detector distances up to 1300 mm may be achieved.

### 2.1. Long-term stability requirements

DIAD operates with a focused diffraction beam. The incoming beam divergence – and thus the divergence of the diffracted beam – is in the range 2Θ = 0.52 mrad. This limits the strain resolution to 3 × 10^−3^ at 2Θ = 10° and 6 × 10^−4^ at 2Θ = 45° in forward scattering. The resolution limit is 1 × 10^−4^ at 2Θ = 135° in backward scattering geometry. Most diffraction experiments will be conducted in forward geometry or at 2Θ angles below 90°, to benefit from the large coverage in reciprocal space and strong forward scattering intensity. Diffraction experiments in backscattering geometry on the other hand provide the highest strain resolution.

The evaluation of the uncertainty at 2Θ = 10° is important as it is close to the minimum scattering angle achievable with the imaging camera in place (Fig. 1 ▸). A transverse detector position instability of 35 µm over a sample-to-detector distance of 300 mm would result in a change of 2Θ around 0.1 mrad at 2Θ = 10° and around 0.06 mrad at both 2Θ = 45° and 2Θ = 135°. These values correspond to an uncertainty in strain of 5.7 × 10^−4^, 7 × 10^−5^ and 1 × 10^−5^, respectively. Hence, in both forward and backward scattering geometry, a 35 µm instability produces errors one order of magnitude below those caused by the divergence. Angles and uncertainties caused by divergence and positioning instability are summarized in Table 1 ▸.

Positioning system instabilities of <35 µm (∼20% of the 172 µm detector pixel size) are therefore deemed acceptable and are set as system specification.

### 2.2. Position repeatability requirements

The required repeatability for the positioning of the diffraction detector followed the same arguments as for the long-term stability and it was considered that 35 µm shifts would not invalidate the diffraction geometry refined during the setup of the instrument.

### 2.3. Vibration requirements

Major sources of instabilities to the detector system were expected from the robot itself (position of the robot arm, servo motors on/off – already partially checked during an initial feasibility study) and the operation of the Pilatus detector (cooling water flow, on-board fan operation) when attached to the robot arm.

Most floor vibrations at Diamond come through in the range below 50 Hz. Based on the permitted shift of 35 µm and a frame rate of 10 Hz, the design frame rate for diffraction experiments at DIAD, the motion target was set to below 35 µm when integrated up to 10 Hz.

The detector itself can operate up to 250 Hz. Assuming all other beamline parameters remain the same, and detector operation at 250 Hz, the motion target would become 35 µm when integrated up to 250 Hz. Operation at 250 Hz at DIAD is currently not possible as the exposure time is flux limited. This case is only considered for completeness as it may become of importance with future source upgrades.

When specifying the vibrational system requirements, it was assumed that instabilities in the incident diffraction beam, specifically beam position instabilities from the upstream optics, would be smaller than any vibrations arising from the robotic detector positioning and were not included in the vibration specification.

## 3. Robot implementation in the DIAD endstation

**Figure 2:**
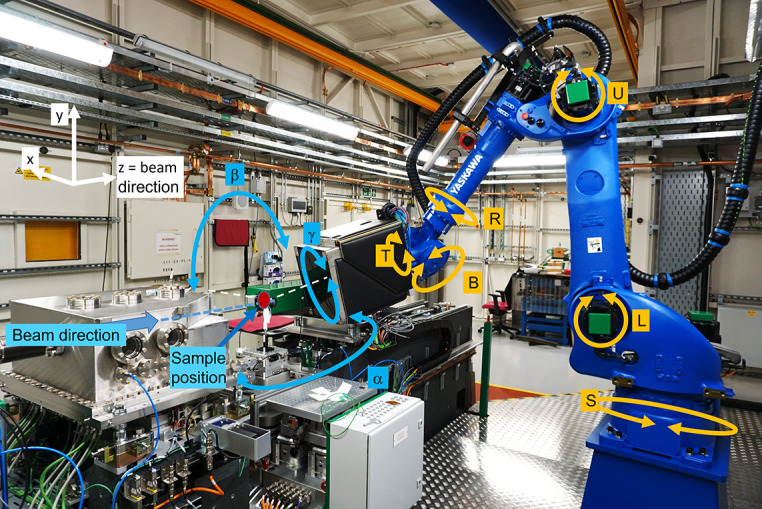
DIAD endstation including diffraction setup with a Pilatus 2M detector mounted on an industrial six-axis robot arm (S, L, U, R, B, T axes), the detector coordinate system (α, β, γ position angles) and the Cartesian laboratory coordination system (*x*, *y*, *z*).

After an initial feasibility study in conjunction with a robot manufacturer, a Yaskawa MH225 six-axis industrial robot arm with a maximum payload of 225 kg was installed in the DIAD endstation to position the detection system (see Fig. 2 ▸). The MH225 series was chosen due to its high payload exceeding the weight of detector (46 kg), detector mount and required cabling (139 kg in total including detector) and its large reach of 2.7 m in the horizontal and 3.3 m in the vertical direction.

The robot is mounted onto a pedestal anchored to the hutch floor. The robot is controlled via a DX200 control unit, has an integral MSU control system (with SIL3/PL=e safety rating) and functional safety zone monitoring (SIL2/PL=d safety rating).

**Figure 3:**
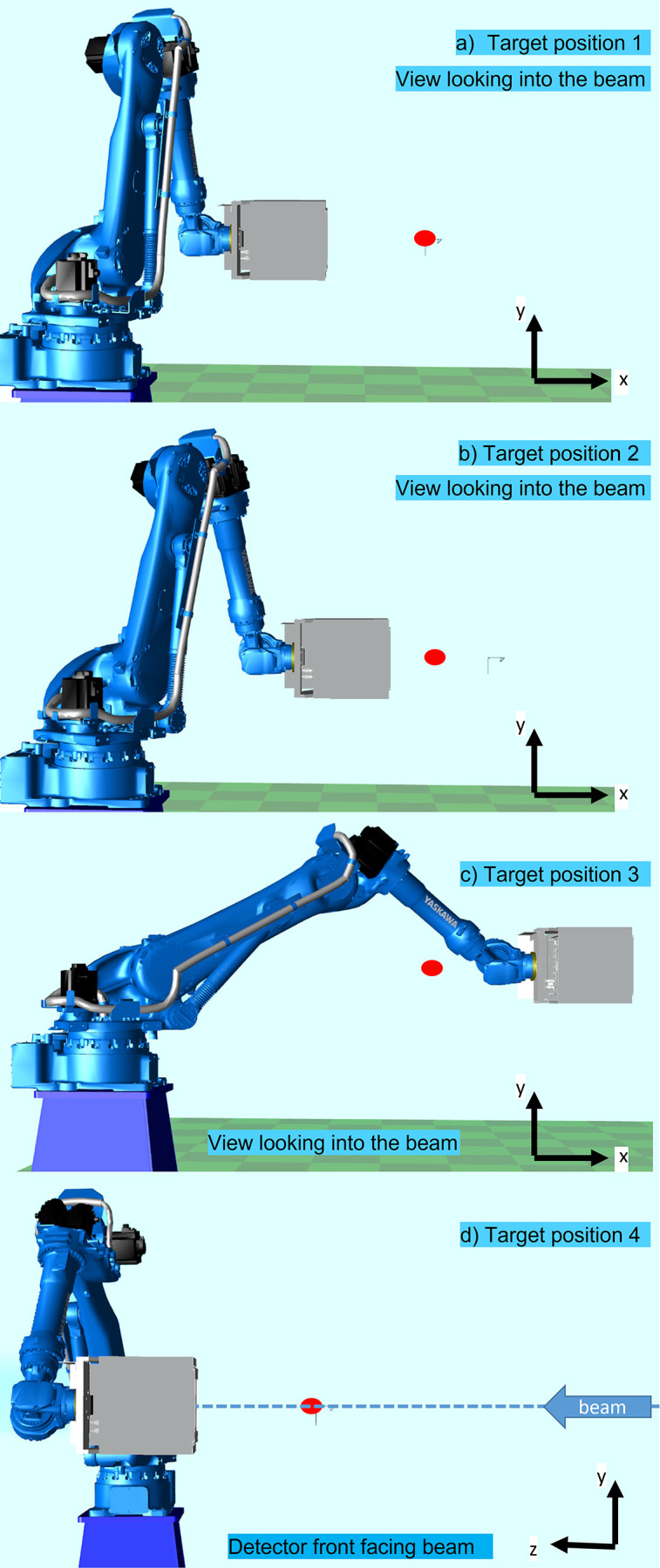
Schematic robot target positioning: positions 1 (*a*) and 2 (*b*) are actual measurement positions, position 3 (*c*) represents the worst case with all robot joints stretches as much as possible, position 4 (*d*) is the positioning for diffraction in the incident X-ray beam.

Positioning of the robot is programmed by converting robot coordinates into spherical coordinates α, β and radius (Fig. 3 ▸). The coordinate system is centred at the intersection of the direct beam and the vertical sample axis. A further coordinate, γ, describes the rotation of the detector around an axis through the centre of and orthogonal to its detection plane with the sample and X-ray beam position as origin. Users can take control of the robot positioning for pre-defined safe positions in automatic operation mode. Advanced positioning needs can be made accessible by manual control of the robot through qualified beamline staff.

The robot can hold position in two different modes: (i) with the servo motors switched on and (ii) with the servo motors switched off and the brakes engaged. The mode with servo motors switched off and brakes engaged is considered the most stable mode and, hence, all considerations around standard operation assume the robot to be in this mode during diffraction data collection.

## 4. Methods

### 4.1. Robot metrology

During measurement sessions, the detector was moved between its home position and four different target positions. The target positions for metrology (see Fig. 3 ▸) were chosen to be as close as possible to actual measurement positions during an experiment, whilst permitting the setup of metrology instrumentation. Care was taken to emulate the operational positions of the robot as much as reasonably possible without compromising the metrology measurements.

The positions used were (1) a target position at α = 90°, β = 0°, radius of 600 mm with most of the individual robot joints being closed/angled, (2) a target position with intermediate stretching of the robot arm at α = 90°, β = 0°, radius of 300 mm, and (3) a fully extended target position emulating the robot being stretched out and the detector being in the backscatter position. Target position 3 is regarded as the least stable position and should represent the worst-case scenario for all measurement positions.

The target position 4 for diffraction measurements was in the direct beam position to enable capturing of full diffraction rings, with the robot coordinates at α = β = 0°, radius set at 330 mm.

A temperature sensor was used to monitor the environment during data capture and found the temperature to be stable to 22 ± 0.1°C. Humidity was not measured.

### 4.2. Accelerometer metrology

**Figure 4:**
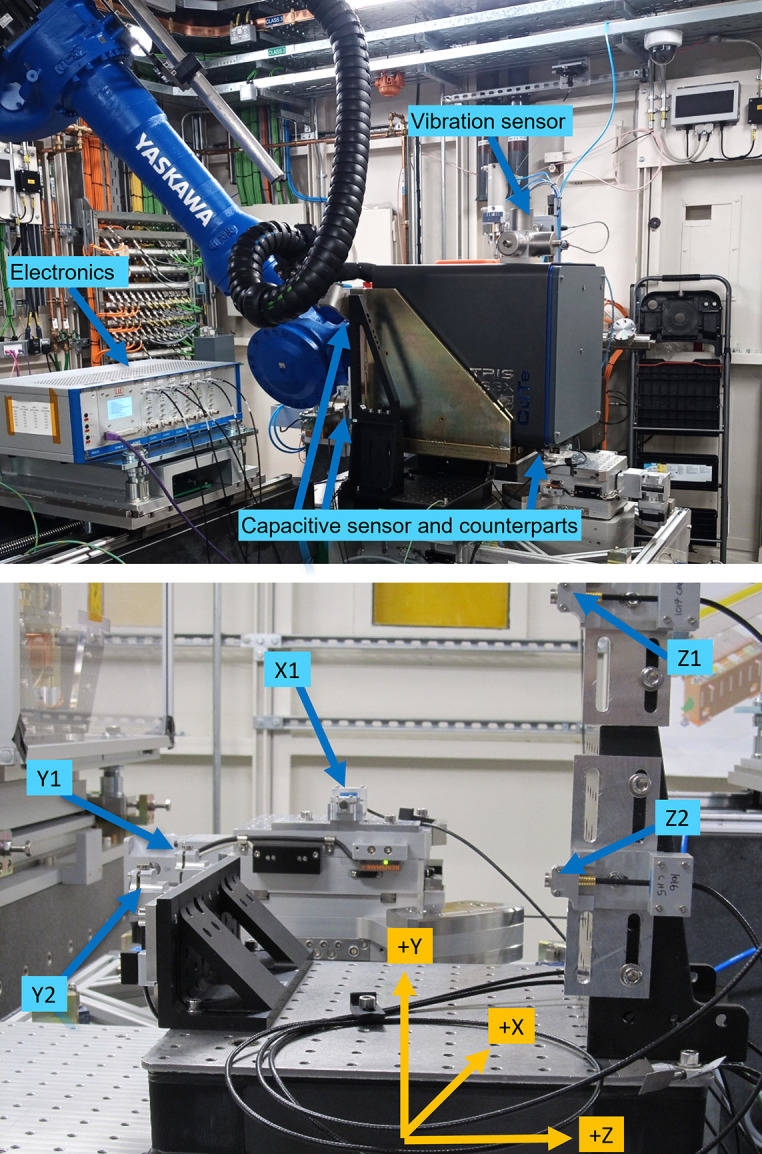
Metrology setup. (Top) Capacitive sensors and vibration sensors positioned around the Pilatus detector. Only three of the five sensors and their actuating counterparts on the detector are visible. (Bottom) Arrangement of capacitive sensors relative to each other.

To understand short-term stability (<1 s) and vibration behaviour of the diffraction detector positioning system, measurements were performed by attaching high-sensitivity accelerometers (Harris & Piersol, 2001 ▸) (PCB Piezotronics 393B31, 10 Vg^−1^) (Christman, undated ▸) (see Fig. 4 ▸). The sensors were connected to a 24-bit NI 9234 sound and vibration card, set to sample at 2 kHz per channel. Two sets of three sensors, each attached perpendicularly to an aluminium block, were mounted within the experimental hutch for reference. The first set was floor-mounted near the base of the robot and used to measure the nominal environmental vibration as a reference; the other set was attached to the top of the Pilatus 2M detector to detect vibrations from the robot arm and/or detector.

The accelerometer data were processed by integrating the collected positional stability data in the 1 Hz and higher frequency range (Smith, 1997 ▸).

### 4.3. Capacitive sensor metrology

A setup of five Micro-Epsilon CSE1 Capacitive Sensors with the NCDT6500 controller was used to measure the linear and angular repeatability, and the long-term stability and associated rotation (see Fig. 4 ▸). Data were collected at a rate of 1.04 kHz, then down-sampled to 260 Hz.

The capacitive sensors were mounted to a frame allowing for two sensors to measure the displacement (average change in two sensors) and rotation (difference between two sensors at a known distance apart), see Fig. 4 ▸ (bottom). Originally, six sensors were envisaged to be used for the measurements; however, one sensor was damaged during experimental preparation. The setup with five sensors was still sufficient to characterize the robot system. The sensors were mounted on the sample-positioning table. The sensors were actuated by brackets mounted on the front and side surface of the Pilatus 2M detector. For the metrology measurements, the sensor stayed stationary whilst the robot/detector moved into position.

This setup was used to measure the long-term stability of the system as well as the system repeatability and short-term vibration measurements.

For long-term stability, the robot was placed in its target position 2 at α = 90°, β = 0°, radius = 300 mm, and kept stationary in this position for 12 h whilst continuously reading out capacitive sensor and temperature data.

Repeatability measurements involved moving the robot from its home position to its target position, switching the servo motors off and engaging the brakes on all joints. Positions from the capacitive sensors were recorded throughout the approach of the actuating elements and recorded all stages of the approach. Position readings from the capacitive sensor were then averaged over a time frame of 3.8 s (1000 position values at a sample frequency of 260.4 Hz). Each measurement was performed five times to reduce the effect of temporary disturbances to the environment, with an arbitrarily chosen wait time of 5 min between each measurement.

### 4.4. X-ray diffraction measurements

To verify that the detector positioning system is sufficiently stable to be used for accurate powder diffraction data collection, a series of diffraction geometry calibrations were conducted on data measured from scattering of a capillary tube filled with lanthanum hexaboride LaB_6_ (NIST SRM660b) powder. To maximize the quality of the powder pattern, the capillary was continuously rotated during the measurement.

**Figure 5:**
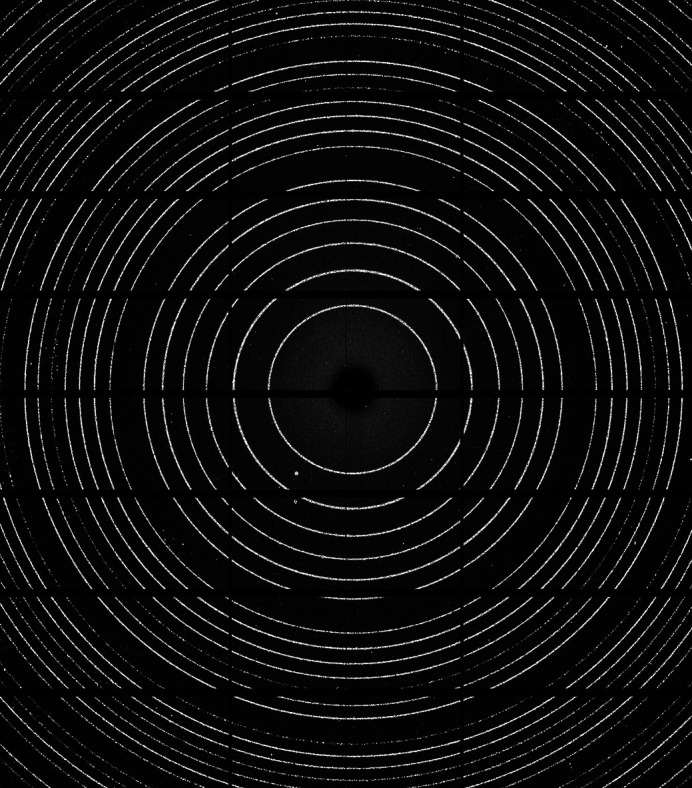
Diffraction pattern collected from a LaB_6_ (NIST SRM 660b) powder calibration standard in forward scatter position used for energy calibration (instrument settings: beam size 2 µm × 15 µm, *E* = 31.52 keV, detector at α = β = 0°, radius of 330 mm).

Data were collected using a focused beam to provide the divergence of 0.52 mrad outlined in the system specification, and the diffraction detector was positioned directly downstream of the incident beam, α = β = 0° at a radius of 330 mm. A representative diffraction pattern is shown in Fig. 5 ▸.

A medium-term measurement lasting 32 min was performed with the detector positioned downstream of the incident beam. After initial positioning, the detector was left for 15 min prior to commencing data acquisition. During the commissioning period of the beamline, it was not possible to perform diffraction measurements over a longer period.

Diffraction studies were then performed by moving the detector to its home position and immediately back to target position 4. After positioning the robot and switching off the servo motors, the hutch was searched and locked up, leading to a delay of ∼105 s before acquiring diffraction patterns. Each acquisition consisted of 30 frames, lasting a total of 150 s. In total, 13 repeats were performed. The results were used to evaluate the short- and medium-term stability as well as the repeatability system.

Diffraction patterns (see Fig. 5 ▸) were calibrated using point-based calibration routines available in *DAWN* (Filik *et al.*, 2017 ▸) using line positions of the first ten diffraction rings, taking a minimum of 500 points per ring. The energy was refined using the first reference diffraction pattern taken in the experiment and subsequently fixed for all following refinements. In the fixed-energy geometry refinement, a total of five parameters were fitted: two angles that describe the orientation of the detector plane relative to a Cartesian coordinate system with the incoming beam direction presenting the *z*-axis; two parameters describing the incident beam position projected to the detector plane; and, finally, the projected distance along the incident beam at which the detector plane is intercepted. These fitted detector parameters were converted to *x*, *y*, *z*, yaw and roll into the laboratory coordinate system.

## 5. Results

### 5.1. Vibration measurements

**Figure 6:**
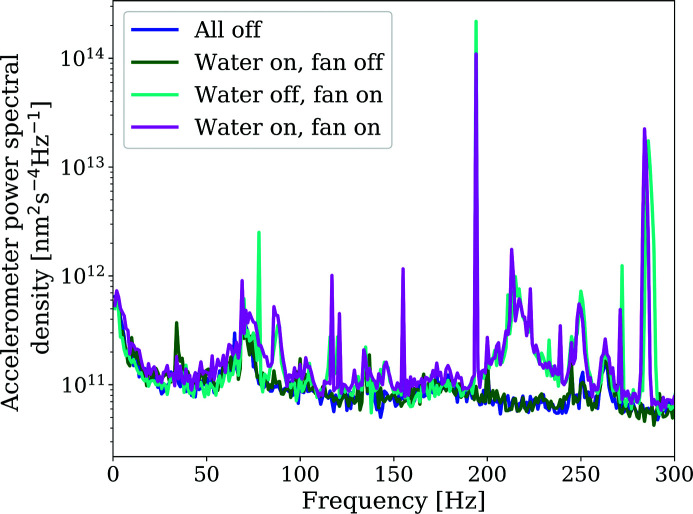
Vibration measurements collected at the diffraction detector under different operation conditions. The accelerometer power shows an increase at a frequency of 220 Hz related to the Pilatus fan operation.

To understand short-term stability (<1 s) and vibration behaviour of the robot arm and detector assembly, measurements using accelerometers were performed (see Fig. 6 ▸).

Input ground motion was minimal (median 40–80 nm RMS). The mechanical susceptibility of the robot mechanics amplifies the vibrations to 5–8 µm RMS median integrated motion over the measurement period and over the whole frequency band. These values are in good agreement with the stability figures measured with capacitive sensors.

Vibrations on the detector remained unaffected upon running cooling water supplied from an external chiller through the detector.

Vibrations from the on-board detector fan led to an increase of vibration in frequencies around 220–230 Hz, as well as at 280 Hz, and several sharp spikes. The difference between fan off and fan on is roughly ∼1 µm RMS, showing that the overall detector stability is dominated by the mechanical amplification of the robot arm rather than the onboard sources.

**Figure 7:**
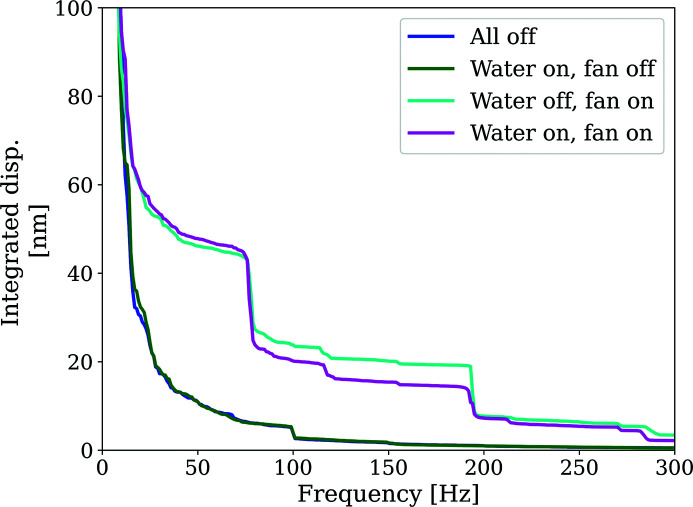
Integrated displacement from accelerometer signals, showing relative contributions of different frequency bands to the total RMS displacement.

Fig. 7 ▸ shows more detail of the contribution of the different conditions to the higher-frequency vibration. Acceleration data were integrated into the frequency domain to first obtain a displacement spectrum, and subsequently accumulated from the higher to the lower frequencies. The data show how the contribution of the fans accounts for ∼40 nm, mainly from the 75–80 Hz and 180–200 Hz spikes visible in Fig. 6 ▸. The remainder of the ∼1 µm difference between fans off and on appears to be linked to the <25 Hz region.

### 5.2. Long-term stability

**Figure 8:**
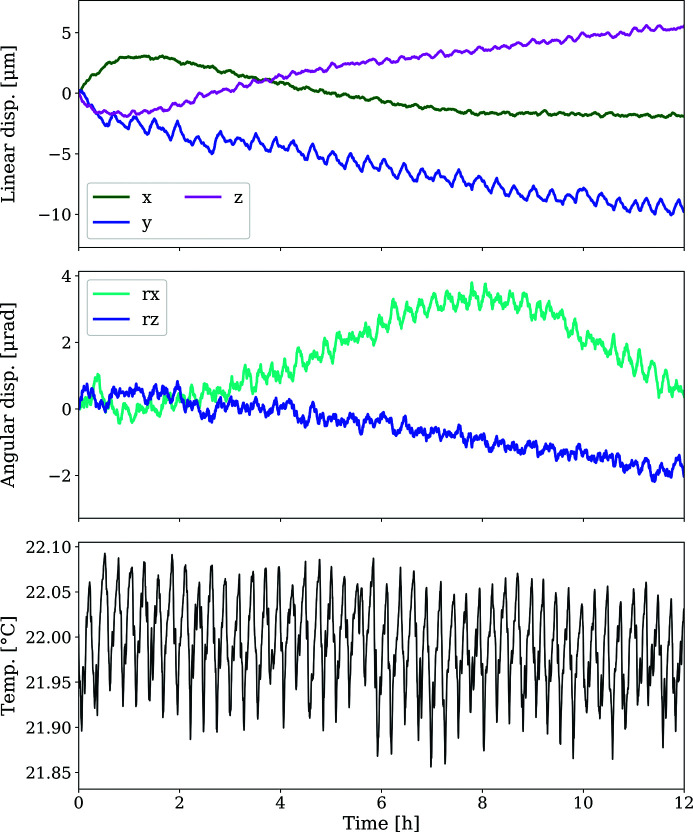
Linear and angular displacement, together with temperature, over a 12 h period under operational conditions for the intermediate position 2 (α = 90°, β = 0°, radius at 300 mm).

Long-term stability measurements over a 12 h period using the metrology setup are displayed (see Fig. 8 ▸). The environmental air-conditioning system cycles between 16 and 20 min and achieves a temperature stability of 22° ± 0.1°C. Medium-term periodic oscillations over one thermal cycle are also present in the capacitive sensor measurement, with linear displacements <1.6 µm and angular displacements <0.9 µrad, respectively. The temperature variation of ±0.1°C correlates directly with these mechanical displacements. It is not possible to deconvolute whether the position changes are due to relative movements of the diffraction positioning system (robot arm, detector mount, detector), the measurements setup of the capacitive sensors themselves, or both.

Over a measurement period of 12 h, minimal drift of the order of <10.1 µm for linear *x*, *y* and *z* displacement and <3.8 µrad for the rotational displacements (rotation about the *x* and *z* axes) were observed (see Fig. 8 ▸).

### 5.3. Repeatability

**Figure 9:**
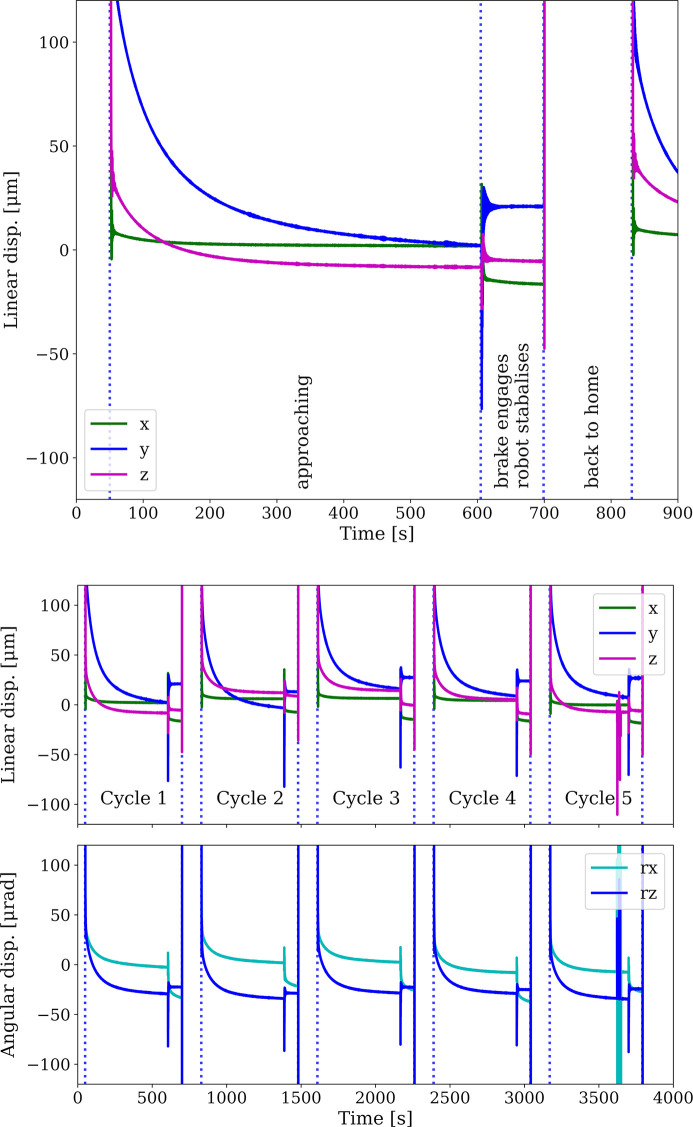
Capacitive sensor readings showing linear displacement during a single positioning cycle (top), and linear and angular displacement plot of multiple approaches (bottom).

Capacitive sensor positions with the robot approaching the target position recording all stages of a single approach are shown (see Fig. 9 ▸), for a single and multiple approaches.

All three different target positions were observed to achieve similar levels of repeatability in all measurement directions. Due to the limited number of repeats performed, only the maximum value of the observed repeatability within all three measurement target positions and all three measurement directions (*x*, *y*, *z*) are reported. The robot returned with a linear repeatability of below 19.7 µm and an angular repeatability of below 16.3 µrad.

**Figure 10:**
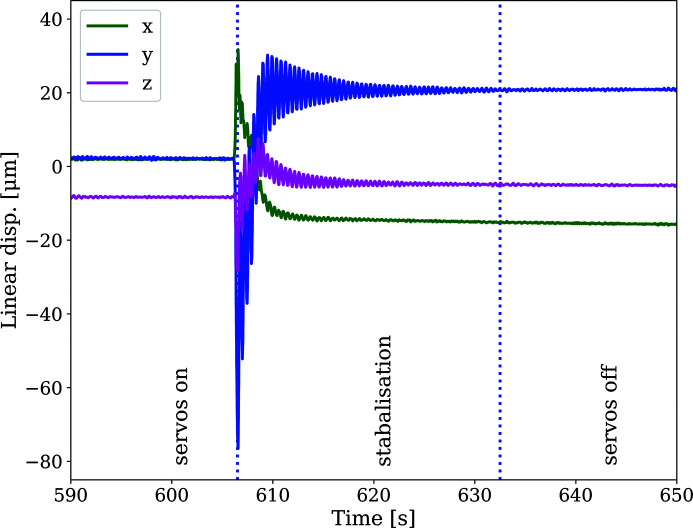
Vibrations measured using the capacitive sensors. The vibration levels with servo motors switched on and switched off are at a similar noise level. The stabilization period after switching the servo motors off is of the order of 30 s.

The vibration stability of the diffraction system has a noise level of 0.18 µm RMS when approaching the measurement position, with the servo motors operating constantly whilst moving the robot. Switching off the servo motors and engaging the brakes resulted in a small position jump. Approximately 30 s after engaging the brake, the position was defined as stable (see Fig. 10 ▸). It is interesting to note that the vibration stability of the system with servo motors switched off has a noise level of 0.2 µm RMS and is hence in a similar range with the servo motors switched on.

### 5.4. X-ray diffraction

X-ray diffraction measurements provided an additional method for quantifying the repeatability and stability in the translational and rotational parameters of the detector geometry. Assessment of the stability of the detector was performed based on values from the fitted scattering geometry of individual images.

**Figure 11:**
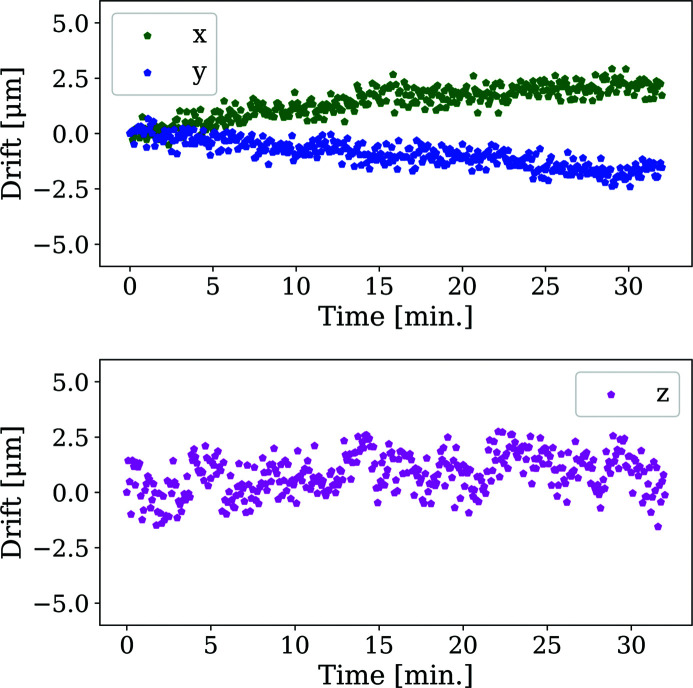
Translational detector parameter drifts in the *x*, *y* and *z* directions measured over a 32 min scan using X-ray diffraction geometry calibration routines available in *DAWN*.

From the medium-term (32 min) stability investigation the fitted parameter drifts are shown (Fig. 11 ▸). It is reassuring to see a general agreement between the magnitudes of the values observed from diffraction and those of the capacitive sensor data previously presented (Fig. 8 ▸).

These data were also used to provide the following estimates of parameter uncertainties arising from the calibration processing: ±1 µm in the *x* and *y* directions and ±2.6 µm in the *z* direction. These estimates come in below those reported for calibration of any individual diffraction pattern: ±3 µm for *x* and *y* and ±3.6 µm for *z*. Angular measurements of the detector taken from the same data showed no systematic variation with time and provided an estimate of the parameter variation to be ±58 µrad. This indicates that the diffraction data cannot resolve angular changes to the same level as the capacitive sensors.

**Figure 12:**
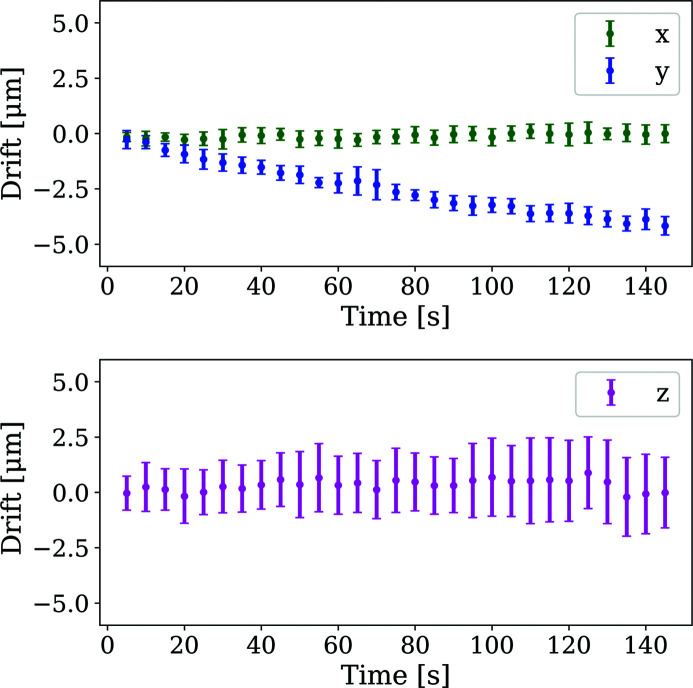
Short-term drifts measured using X-ray diffraction. The time shown is relative to the first frame for each movement cycle, *i.e.* around 1 min 45 s after the robot has completed the movement.

Assessment of the short-term stability of the detector was performed based on the scattering geometries for 30 frames of the 13 detector movements. Drifts in the translational detector parameters post-positioning were measured relative to the first frame for each scan to remove any differences due to repeatability (see Fig. 12 ▸) and the error bars shown represent the certainty on the mean to the three standard deviation level from the 13 repeats.

Whilst *z*-direction changes showed the largest variation across repeats, represented by the large error bars, when considered alongside the results of Fig. 11 ▸, none of the grouped means vary significantly from zero, indicating that on average, after positioning, the distance from the detector plane to the sample remains constant. Similarly, drifts in the *x* direction remain negligible. A drift in the *y* direction of approximately 4 µm builds up over the measurement period of 150 s. The consistency of this value between repeated movements indicates that this movement is real, but it is difficult to uniquely ascribe an underlying cause.

Repeatability estimates were made from assessment of the calibrated geometry variations observed from the first frames of data collected for each of the 13 repeats; an estimate of repeatability is determined to be 17 ± 5 µm in *x* and −9 ± 5 µm in *y* and 15 ± 6 µm in *z*. In direct comparison with the capacitive sensor measurements for the translational repeatability, these measurements are in good agreement; however, they also demonstrate that such repeatability variations are detectable in the diffraction pattern.

## 6. Discussion and future perspective

### 6.1. Vibration measurements

Vibrations affecting the positioning of the detector are small. The stability figures obtained from acceleration sensors are in close agreement with the figures measured with capacitive sensors, in the range 5–8 µm RMS median integrated motion over the measurement period.

Vibrations introduced by the detector services (external cooling water supply, detector on-board cooling fans) were investigated; the main contributor (∼1 µm RMS) was found to be due to the cooling fans whereas contributions from the cooling water flow were negligible.

The origin of the sharp peaks at ∼190 and ∼280 Hz is unclear but the narrow band nature of those peaks suggests a sharp mechanical resonance. It is outside the scope of this manuscript to try to understand the exact nature of these resonances. So far, the experimental diffraction data did not show signs of unwanted vibrations.

### 6.2. Thermal stability

A baseline comparison for any stability measurements is the stability of the environment in which the diffraction detector positioning system is located, with temperature variations being the main source of concern. The hutch temperature cycles around 22°C with variations of ±0.1°C over a 16–20 min time frame. This temperature variation correlates directly with linear and angular displacements in the range ∼1.6 µm and 0.9 µrad, respectively, obtained from the long-term capacity sensor metrology measurements. For a diffraction experiment, at a sample-to-detector distance of 300 mm, the 1.6 µm position variation would be expected to result in a strain variation of 3 × 10^−5^, 3 × 10^−6^ and 6 × 10^−7^ measured at scattering angles of 2Θ = 10°, 45° and 135°, respectively. These are substantially below the resolution limit.

### 6.3. Stability after initial robot positioning

Another aspect of the positioning system is the question of whether a wait time after positioning robot and detector is required before diffraction data can be acquired. This information was obtained from cap sensor measurements. The robot reaches steady state approximately 30 s after switching off the robot servo motors and engaging the brakes (see Fig. 10 ▸). During the robot commissioning phase, it was not possible to obtain diffraction data directly after positioning the robot, hence a direct comparison with time series of the diffraction pattern during these early stages was not possible.

Diffraction data are, however, available from ∼1 min 45 s after the detector positioning. Whilst the linear positioning in the *x* and *z* directions remain very stable, a drift in the *y* direction of up to 4 µm over a 150 s time frame builds up (see Fig. 12 ▸).

It is interesting to note that this drift is in the vertical direction downwards. There are multiple possible explanations for this behaviour: (i) a slow vertical downwards drift of the robot arm due to gravity, (ii) environmental factors such as temperature changes within the hutch after closing the hutch door, and (iii) a movement of the incoming beam caused by warm-up of the Kirkpatrick–Baez optics.

Evidence for a vertical drift is visible in the capacitive sensor measurement, accumulating 0.3 µm after 150 s, 1.6 µm after 20 min, and 10.1 µm after 12 h. In addition, temperature variations due to hutch closure are seen, but remain limited to below 1.5 µm as discussed above. Evidence for or against beam movement due to shutter opening is not available, hence beam movement cannot be excluded as a source of the vertical movement seen in the diffraction data. As the influence of varying temperature and beam movement is independent of the robot arm but caused by other factors not related to the robotic positioning, they are not considered as impacting on the robot stability itself further discussed here. The stability of the robot positioning system is best based on the metrology results (mechanical and X-ray-based), with the result that a wait time of 30 s after switching off the robot servo motors should be sufficient to avoid any negative impact from the position change onto the diffraction data.

### 6.4. Long-term stability over 12 h

The long-term stability of the detector positioning system (<10.1 µm linear stability, <3.8 µrad angular stability) is shown to be well within the given specification of <35 µm. The periodic oscillations in positioning (<1.6 µm) due to fluctuations in hutch temperature are visible throughout the collected data. In addition to the medium-term oscillations (16–32 min), drift of systems away from the starting positions becomes visible.

### 6.5. Repeatability measurements

The measured repeatability from capacitive sensors is below 19.7 µm linear and below 16.3 µrad angular; the repeatability measured via diffraction measurements is 22 µm linear and 59 µrad angular repeatability.

There is good agreement between the linear repeatability obtained by capacitive sensors and from diffraction. The capacitive sensor data are the most accurate representation of the repeatability.

The translational repeatability of 19.7 µm at a sample-to-detector distance of 300 mm would provide a variation in strain of 3.7 × 10^−4^ when measured at a scattering angle of 2Θ = 10°. Elastic strain measurements in materials require strain measurement uncertainties between 1 × 10^−4^ and 1 × 10^−5^ (Withers *et al.*, 2001 ▸; Withers, 2004 ▸). Strain variation from detector repositioning is, therefore, on the edge of being plausible to avoid the need for recalibration after moving the detector and would be suitable for phase identification measurements where less precision is required. In fact, if the reflection of interest scattered at an angle of 2Θ = 45°, the strain uncertainty would fall within the required range.

From our data, the calibration precision is clearly not limited by constraints imposed by the divergence of the focused beam. During the calibration routine, peak fitting in the radial direction of the intensity distribution of a peak is performed. For an ideal powder, the radial intensity profile obtained with a divergent beam will be steady, and consequently a peak location can be determined to a certainty well below the width of the profile. Moreover, from including many points within each reflection, a detector calibration including partial or complete Debye–Scherrer rings can determine the beam centre location in this geometry to sub-pixel precision.

For experiments which require an intermediate diffraction resolution, repositioning to multiple locations is possible, assuming instrument calibration remains valid and other beamline parameters (energy, beam size) are unaltered. This is a big advantage for diffraction experiments where the robot needs to be moved out of its position to allow access to the sample and/or experiments involving multiple detector positions. Whether a calibration is necessary after every detector move will depend on the exact nature of the experiment and the required strain resolution.

### 6.6. Advanced diffraction measurements

It is interesting to note that the vibration noise levels within the diffraction positioning system have comparable levels of around 0.2 µm RMS, independent of whether the servo motors are switched off/brakes engaged or the servo motors running. These results were not expected, as the preliminary feasibility tests without robot calibration/optimization indicated much higher noise levels with the servo motors switched on. With the impact of the servo motor operation removed, it allows re-thinking the use of the diffraction positioning system.

Whilst it was never envisaged to operate the positioning system in a scanning mode, with the detector moving around the sample position in a dynamic manner whilst collecting diffraction data, this mode may be possible to achieve from a vibration point-of-view. Depending on further pre-characterization and feasibility checks, it may be possible to carry out advanced trajectory measurements, giving the user community an opportunity to explore new diffraction techniques.

## 7. Summary

The DIAD beamline has successfully integrated an industrial-size robot arm into its endstation with the aim to position a Dectris Pilatus 2M CdTe detector. Detector, detector mount and cables have a combined weight of 139 kg and must be kept stable in position to <35 µm to guarantee collection of good quality diffraction data.

Metrology measurements were performed to verify the system stability and repeatability. Independently of the position of the detector in the quarter-sphere around the sample position, the detector can be kept stable to below 10.1 µm for linear translations and below 3.8 µrad over a 12 h period. Position variations of −1.5 µm arise from temperature variations within the experimental hutch and further variations of −1 µm can be contributed to vibrations from the on-board cooling fans of the Pilatus detector.

The detector can be re-positioned to the same location with a repeatability of better than 19.7 µm and 16.3 µrad.

Whilst the metrology data alone give a good indication of the stability of the robot-based positioning system, the actual deciding factor is the quality of diffraction data obtained using this system. It has been demonstrated that, under optimal scattering conditions with the detector positioned 330 mm downstream of the sample, instrument scattering geometry can be refined to approximately 1 µm in the *x* and *y* directions and 3 µm in the *z* direction. Diffraction is not, however, an optimal way to measure rotation of the detector for the instrument with the orientation resolution estimated around ±58 µrad.

The robot positioning system keeps the detector stable in position, so that the geometry calibration of the setup remains valid over several hours. The calibration is also dependent on other factors (environmental parameters, beam stability *etc*.). The validity of the calibration will depend on the required experimental resolution, with experiments that require higher resolution also being required to re-calibrate more frequently.

The repeatability of the robot positioning of the detector is within the specification. The peak position resolution required from diffraction data will determine whether re-positioning is possible without compromising the data quality. For the highest strain resolution requirements, re-positioning the robot will most likely exceed the expected strain resolution and is therefore not recommended for the DIAD setup. Re-positioning to one or multiple positions may well be possible for lower resolution requirements.

The exact choice of positioning and operation of the robot are dependent on the needs of the experiment and need to be considered as part of the experimental setup and may change depending on the required resolution.

## Figures and Tables

**Table 1:** Strain resolution in forward and backward scattering positions at important scattering angles for a detector at 300 mm from the sample

Position	Scattering angle, 2Θ (°)	Strain resolution due to beam divergence	Strain resolution due to 35 µm position instability
Forward scattering†	10	3 × 10^−3^	5.7 × 10^−4^
Forward scattering	45	6 × 10^−4^	7 × 10^−5^
Backward scattering	135	1 × 10^−4^	1 × 10^−5^

† Smallest achievable diffraction angle, closest position to the imaging camera.
